# Chronic Prostatitis/Chronic Pelvic Pain Syndrome: A Disease or Symptom? Current Perspectives on Diagnosis, Treatment, and Prognosis

**DOI:** 10.1177/1557988320903200

**Published:** 2020-01-31

**Authors:** Jianzhong Zhang, ChaoZhao Liang, Xuejun Shang, Hongjun Li

**Affiliations:** 1 Department of Urology, Peking Union Medical College Hospital, Peking Union Medical College, Chinese Academy of Medical Sciences, Beijing, China; 2 Department of Urology, The First Affiliated Hospital of Anhui Medical University, Hefei, China; 3 Reproductive and Genetic Laboratory, Jinling Affiliated Hospital of Nanjing University School of Medicine, Nanjing, Jiangsu, China

**Keywords:** Chronic prostatitis/chronic pelvic pain syndrome, clinical evidence, associated symptoms, diagnosis, treatment

## Abstract

Definitive diagnosis and selection of effective treatment for chronic prostatitis/chronic pelvic pain syndrome (CP/CPPS) are frustrations encountered frequently by urology care providers in their practice. Knowledge of etiology and pathophysiology is not sufficient and therapeutic guidelines have not yielded acceptable outcomes and prognoses for both patients and care providers. The authors present updated perspectives on CP/CPPS, including definition, diagnosis, treatment, and prognosis, based on literature review and clinical experience. A key point is to shift the diagnostic and therapeutic focus from a single entity of disease toward associated symptoms of CP/CPPS. An individualized multimodal treatment approach to cope with the course of the disorder is proposed. Communications and personal/family/community supports are emphasized as an important component in the therapeutic regime and rehabilitation of patients with CP/CPPS. The purpose is to improve comprehension on CP/CPPS and to help care providers and patients to achieve the goal of medical intervention—relieving associated symptoms of CP/CPPS and improving the quality of life.

Chronic prostatitis (CP) is a male health issue commonly seen in adulthood. The best treatment option for this disorder is uncertain. Based on the consensus of the National Institutes of Health (NIH) published almost two decades ago, it is defined as chronic bacterial prostatitis (CBP) and chronic prostatitis/chronic pelvic pain syndrome (CP/CPPS; Krieger et al., 1999, 2002). The etiology and pathophysiology of CP/CPPS remain largely unknown. Varied prevalence estimates of CP/CPPS from 8.4% to 25% have been published in different continents (Habermacher et al., 2006; Liang et al., 2009; Zhang et al., 2019); 35%–50% of the male population were reported to be likely affected by associated symptoms during their lifetime (Gao et al., 2015; Krieger et al., 2008; McNaughton Collins et al., 2001; Pavone-Macaluso, 2007, Zhang et al., 2019). Differences in reported prevalence of CP/CPPS are associated with many factors including genetics, ethnic groups, cultural backgrounds, educational levels, psychological status, lifestyles, food habits, health status, family relationships, and other socioeconomic conditions. The knowledge gaps in the mechanism of CP/CPPS occurrence plus uncharacteristic and variable clinical presentations have created a formidable obstacle for clinical epidemiological investigations of CP/CPPS.

The lack of insight into CP/CPPS and of a clear clinical definition has led to unsatisfactory management of patients in clinical practice. There are no evidence-based studies with randomly designed, large cohorts and long-term follow-up on the effects of medical intervention on CP/CPPS patients and prognosis of the disease. A major difficulty in medical treatment of patients with CP/CPPS is extended time of therapy, uncertainty of outcomes, and relapse of symptoms, posing a significant impact on health care resources and family well-being. This causes us to rethink: Is medical intervention the only way to solve this problem? How could CP/CPPS be managed cost-effectively through different approaches? What is the role of psychological and behavioral therapies in the treatment regime of CP/CPPS? What types of support do the patients need from sexual partners, family, and peers in social circles? What can lifestyle adjustment do for symptom relief of CP/CPPS? How important is the patient’s knowledge of CP/CPPS in treatment and rehabilitation? These questions have attracted more interest from medical and social sciences as well as public and social media now than ever before. The current perspectives on CP/CPPS are to provide updates and analyses on concept evolution and management of CP/CPPS to stimulate further discussions on the subject. Some of the opinions will improve clinical comprehension and patient/family education on CP/CPPS and promote individualized and symptom-orientated multimodal therapies for CP/CPPS. A critical point is to propose a definition of CP/CPPS from a clinical point of view that will form the basis for better diagnosis and treatment in the near future.

## Definition: A Set of Symptoms Rather Than an Entity of Disease

The debates about the definition of CP have been ongoing for decades (Doiron et al., 2019, Jhang & Kuo, 2017). The latest version of the NIH consensus classification proposed that the diagnosis of CP/CPPS relied on the presence or absence of leukocytes in expressed prostatic secretions (EPS), urine after prostatic massage (VB3), or seminal fluid analysis (Krieger et al., 2002). These laboratory evidence-based criteria added almost twice as many patients into the category of CP/CPPS as the traditional concept of nonbacterial prostatitis. The heterogeneity of the disorder itself creates major difficulties in defining CP/CPPS (Polackwich & Shoskes, 2016). Some patients diagnosed with CP/CPPS have suffered from associated symptoms clinically without laboratory evidence, while associated symptoms of patients with laboratory-evidenced CP/CPPS were not always alleviated by selected therapy targeted to laboratory findings, and vice versa. Shoskes divided CP/CPPS into six subtypes in acknowledgment of clinical presentation, subjective description, and psychosocial background, that is, urinary (U), psychosocial (P), organ specific (O), infection (I), neurologic/systemic (N), and tenderness (T), simplified as UPOINT (Shoskes et al., 2010). This classification of CP/CPPS has begun to shift the focus to a symptom-orientated diagnosis and treatment model (Samplaski et al., 2012). A standard terminology for CPPS was published by a special working group of the International Continence Society (ICS). Upon clinical presentation, different types of CPPS were categorized into nine clinical domains, that is, lower urinary tract, female genital, male genital, gastrointestinal, musculoskeletal, neurological, psychological, sexual, and comorbidities (Doggweiler et al., 2017). This terminology emphasized symptomatic subtypes of CPPS and potential factors associated with the pain, providing further support for symptom-orientated diagnosis and treatment.

We propose that CP/CPPS is reasonably defined as a set of associated symptoms rather than as a single entity of disease. Four elements are essential to diagnose CP/CPPS, including (a) symptoms occurring in perineal and/or low abdominal region, (b) infection and/or inflammatory changes of the prostate with laboratory evidence of abnormal findings, (c) clinical presentations (mainly pain and discomfort) derived from or associated with the prostate and lower urinary tract, and (d) symptoms appearing more or less after an inducible cause with varied incubation times. A set of associated symptoms include, but are not always limited to, pain and/or discomfort with urinary alteration, abnormal secretion from the urinary tract, and ejaculation pain, and so forth. Each individual has his own main complaint and several other symptoms in combination (Krieger et al., 2008). The symptoms usually fluctuate but continue for at least 3 months. Using this definition of CP/CPPS, the urology care providers will focus on resolving associated symptoms individually by selection of therapeutic approaches while looking for relevant evidence to support the diagnosis during the course (Guan et al., 2015).

## Diagnosis: Clinical Comprehension With a Combination of Symptoms and Evidence

The lack of a known etiology has made a definitive diagnosis difficult. Laboratory examinations have given reference values if there are positive findings. Studies suggest that leukocyte and bacterial counts in EPS are not always correlated with the severity of symptoms (Nickel et al., 2003). Thus, some classic methods (such as the pre- and post-prostate massage culture test) need to be reevaluated clinically for a confirmative diagnosis of CP/CPPS. In a longitudinal study for 4 years, Nickel et al. investigated the relationship between an early histological change of prostate inflammation and later onset of CP/CPPS and reported that inflammatory changes of the tissues did not increase the risk of CP/CPPS (Nickel et al., 2003, 2017). A group of potential candidate biomarkers for urological CPPS (UCPPS) have been screened and examined; none of those biomarkers could separate the patients with UCPPS from control groups definitely (Dagher et al., 2017). These facts and challenges endorse the need to reconsider the diagnosis of CP/CPPS in a clinical setting while continuous efforts are put forth for its etiology, standardization for terminology, and development of effective therapy (Doggweiler et al., 2017; Kessler, 2017).

The phenotype-based diagnosis of CP/CPPS (Shoskes et al., 2010) and UPOINT (Hao et al., 2011; Li & Kang, 2016) are highly recommended. UPOINT is more meaningful for urology care providers in evaluating associated symptoms of CP/CPPS than the evidence-based diagnostic guideline. Associated symptoms may be linked to other disorders such as varicocele, irritable bowel syndrome, and melena (Li et al., 2002; Lotti et al., 2009; Pavone et al., 2000; Vicari et al., 2011, 2014). Those phenotypes need to be ruled out in the diagnosis of CP/CPPS. Based on the authors’ experience, the exclusive diagnostic algorithms of CP/CPPS start with low abdominal pain and/or abnormal urination (so-called alarming signs), prostate palpating, and two basic laboratory examinations, that is, urine macroscopic test and ultrasound of low abdomen region. These examinations give the care providers a clue to exclude abnormal results that may be caused by other disorders such as tumors, tuberculosis, urolithiasis, nephropathy, and bladder infection. Age is another phenotype to consider at initial visits of patients with associated symptoms. Patients under 50 years of age are more likely to suffer from CP/CPPS than those over 50 years whose symptoms are likely caused by other diseases. Prostate-specific antigen (PSA) is usually ordered for patients >50 years old in the exclusive diagnostic procedure for CP/CPPS.

**Figure 1.**
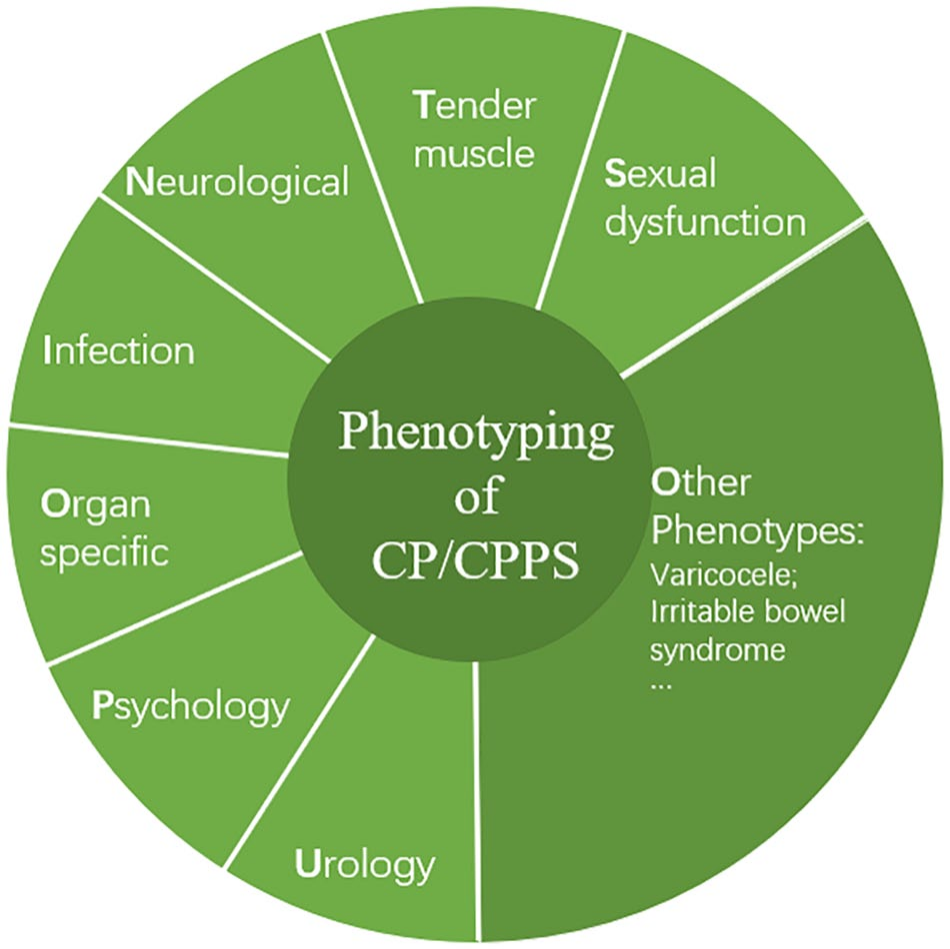
Phenotypes possibly associated with chronic prostatitis/chronic pelvic pain syndrome (CP/CPPS).

The terminology of CPPS published by ICS is usually used for measuring scales and locations of pain (pain mapping; Doggweiler et al., 2017). The NIH-Chronic Prostatitis Symptom Index (NIH-CPSI) and the consensus guideline by the Prostatitis Expert Reference Group are also useful instruments for initial evaluation of symptom severity and follow-up of treatment results of CP/CPPS (Collins et al., 2001; Litwin et al., 1999; Rees et al., 2015). A taxonomy provided by the International Association for the Study of Pain (IASP) recommended that pain in the pelvic region should be considered as a multidisciplinary issue including urologic, gastrointestinal, musculoskeletal, neurologic, and/or rheumatologic etiology with psychosocial aspects (Doggweiler et al., 2017). In addition, the European Association of Urology (EAU) guidelines subdivide chronic pelvic pain (CPP) into conditions that are associated with pain and those with nonpain syndromes (Engeler et al., 2019). The latter have well-recognized pathology (e.g., infection, neuropathy, or inflammation), whereas the former do not have a clear etiology. Although the EAU classification deals primarily with urological disorders, it can be applied to all conditions associated with pain perception within the pelvis. Figure 1 summarizes phenotypes possibly associated with CP/CPPS.

## Treatment: Individualized Phenotype-Directed Multimodal Therapy Targeting Associated Symptoms

As long as an initial diagnosis of CP/CPPS is established for associated symptoms with laboratory changes, clinical evidence, and phenotype, selection of treatment protocol should be individualized without delay. There are many approaches available in current clinical practice, including α-blockers, antimicrobial and anti-inflammatory medicines, neuroleptics, antidepressants, physiotherapy, acupuncture, extracorporeal shock wave therapy (ESWT) phytotherapy, and others for pain relief (Agarwal & Elsi Sy, 2017; Al Edwan et al., 2017; Chang et al., 2017; Franco et al., 2018; Hu et al., 2019; Rees et al., 2015; Shoskes, 2003; Yuan et al., 2019; Zhang et al., 2017). In a comprehensive review of 28 published randomized controlled trials (RCTs) from 1998 to 2014, Magistro et al. summarized trial results with antibiotics, α-blockers, anti-inflammatory and immune-modulating substances, hormones, phytotherapeutics, neuromodulatory drugs, and physical treatment and concluded that none of the efficient monotherapeutic options was beneficial to all patients with CPPS (Magistro et al., 2016). The psychosocial burden of the associated symptoms of CCPS on patients has been studied (Brunahl et al., 2017). Evidence indicated that psychological consultation has a clear benefit for patients while confirming a diagnosis of CP/CPPS (Sung et al., 2014). A case was given as an example of clinical experience. CP/CPPS was diagnosed without laboratory abnormality in a 42-year-old patient and treated with different antibiotics and analgesics for 2 years. His symptoms had not improved but were aggravated by psychological distress and anxiety, upsetting his life and work significantly. Based on chart review and analysis, we had discontinued antibiotics and analgesics and then used suggestive therapy, placeboes (vitamin E and the Chinese herb LiuWei DiHuang pill), plus explanation about CP/CPPS as the treatment approach. The patient’s symptoms were relieved remarkably and they did not bother him in his daily life after 10 weeks. The Chinese herb pill has no therapeutic effect on CP/CPPS at all. However, the patient was a strong believer in traditional Chinese medicine, which helped in his symptom relief. Thus, an understanding of the patient’s personality and characteristics is also beneficial to the care providers in the management of CP/CPPS. Monotherapeutic protocol seldom yields satisfactory results for both patients and care providers at present likely because of the heterogeneous nature and multifactorial pathophysiology of CP/CPPS. Thereupon, use of large quantities of multiple drugs is not recommended for the management of CP/CPPS from a pharmacoeconomic point of view. With its various clinical presentations, a multimodal therapeutic approach addressing the clinical phenotypic profile of each patient was recommended as the best evidence-based management of CP/CPPS (Magistro et al., 2016; Smith, 2016).

Multimodal and individualized management is a preferable choice for patients with CP/CPPS clinically. This management regime should be selected with caution in order to achieve the best clinical practice for managing patients. Several critical points are addressed as follows: (a) An empirical multimodal protocol or a single-agent therapy (with solid pathological and laboratory evidence) should be initialized right after a diagnosis of CP/CPPS and is mostly acceptable to the patients who visit a clinic. (b) Drugs with virulent effects and potential side effects and apparatus with unproven clinical benefits should be avoided, such as combined antibiotics, transcutaneous electrical nerve stimulation, urethral infusion therapies, and surgeries (Lee et al., 2003; Nickel et al., 2001; Nnoaham & Kumbang, 2008; Thakkinstian et al., 2012). (c) Some mild analgesia targeting the central nervous system and antianxiety/depression drugs (duloxetine, fluoxetine, sertraline, amitriptyline, etc.) might help to alleviate associated pain (Furlanetto et al., 2013; Giannantoni et al., 2014; Kutch et al., 2017; Shulyak et al., 2019; Xia et al., 2011; Zhang et al., 2017). (d) Sufficient information about special diet, lifestyle, social environment, and sexual habits should be obtained and advice should be given to the patients for these modifiable conditions, which may alter the symptoms and the severity of pain (Chen et al., 2016). (e) It is unnecessary to make a clear distinction between the category IIIA (inflammatory CP/CPPS) and IIIB (noninflammatory CP/CPPS) when applying a treatment protocol since there is a lack of evidence to support its clinical value during the treatment (Sung et al., 2014). (f) Importantly, monitor individual response to the selected medical intervention closely. This is an important step in confirming the diagnosis of CP/CPPS since the symptoms of patients are not only altered by the initial selected treatment but also influenced by their socioeconomic background, other medical conditions, and awareness (Nickel, 2002).

A treatment of CP/CPPS is considered successful if specific measures (see Prognosis section) provide good results. Some of these measures are recommended to be repeated in the next two consecutive clinical follow-ups at 2-month intervals. Based on clinical observation and follow-up in daily practice for many years, phytotherapy targeting the symptoms in combination with simple analgesics and/or α-adrenergic antagonists (if voiding is present) is recommended as the first line of treatment for a good clinical outcome of CP/CPPS. Use of antibiotics has become less and less important in achieving therapeutic benefits for patients with CPPS (Doiron et al., 2019). Efforts and consultations by a multidisciplinary team are highly recommended, including urology care providers, pain control specialists, physiotherapists, psychologists, cognitive behavioral therapists, and sex health counselors.

## Prognosis: Relieving Symptoms and Improving Quality of Life Instead of “Curing”

The relief of associated symptoms and improvement of quality of life is a new treatment goal, coping with changes of diagnostic and management approaches on CP/CPPS (Jhang & Kuo, 2017). Associated symptoms of CP/CPPS need to be controlled to such a level that there are no or negligible negative effects on the daily life of individuals physiologically and psychosociologically. The prognosis of CP/CPPS should be measured with “realistic facts”, that is, many mild cases of CP/CPPS (lower scores on CPSI prior to treatment) are completely satisfied with treatment outcomes even though their CPSI scores never reach zero (Reichard et al., 2015). The prognostic measure will change the present definition of treatment end point for CP/CPPS, that is, eradication of etiological/pathological changes and all associated symptoms. This is an essential transformation for both the care providers dealing with the disorder and patients suffering from CP/CPPS.

Specific measures for good treatment prognosis of CP/CPPS should include (a) complete resolution or substantial relief of the subjective symptoms with negative findings in EPS (including bacterial culture); (b) significantly improved prostate palpation and/or no tangible pain at all; (c) normal segmental urine examination; and (d) recommencement of habitual sex that patients and their sexual partners preferred and no ejaculation pain. Aggressive therapeutic protocols have seldom yielded very satisfactory prognosis for patients who had completely recovered from CP/CPPS (Koh et al., 2014). Combined therapy is recommended including reasonable regime of treatment, good dietary habits, psychological consultation, and supportive social and family environment in order to achieve a good prognosis and avoid recurrence (Giannantoni et al., 2014; Koh et al., 2014; Liang et al., 2009; Zhang et al., 2015). Time seems an important factor in symptom relief for CPPS. Most patients with CP/CPPS have achieved clinical relief of associated symptoms even without treatment in some cases and lead a normal daily life with time (Zhang et al., 2019).

## Communication and Support: An Important Component for Treatment and Prognosis of CP/CPPS

**Figure 2.**
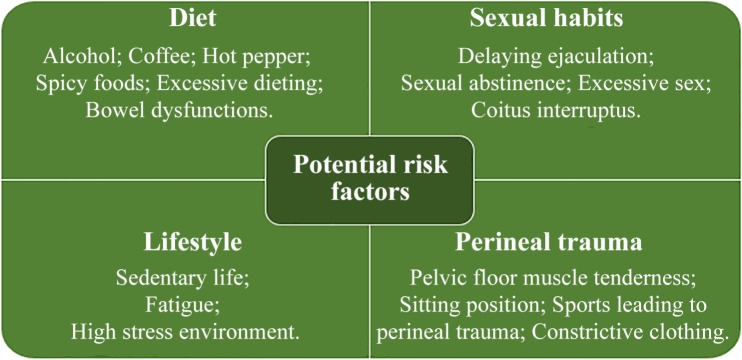
Potential risk factors associated with chronic prostatitis and chronic pelvic pain syndrome.

CP/CPPS is a chronic disorder of males. Not only do individuals suffer from pain physiologically and psychologically but there is also a long-term impact on their family life. Treatment regime and rehabilitation require sometimes an extended period of time. Both the care providers involved and patients should be aware of the facts. Communication, education, and peer/family/community support are important for a successful intervention. There must be effective communication and exchange of patient information between physicians in general practice and urology care providers involved in the multidisciplinary therapeutic team. Education is an essential component for individuals and their sexual partners to understand and accept the concept changes in current perspectives on CP/CPPS. A clear explanation of the nature of the disorder, chronic pain cycle, treatment options/courses, and possible outcomes should be available to the patients and their sexual partners if applicable (Rees et al., 2015). Communication between patients and their sexual partners is highly suggested and involvement of the patient’s sexual partner is encouraged from the beginning of medical intervention. Gallo et al. listed 13 potential risk factors associated with CP/CPPS including diet (alcohol, coffee, hot pepper and spicy foods, excessive dieting, bowel dysfunctions), sexual habits (delaying ejaculation, sexual abstinence, excessive sex, coitus interruption), lifestyle (sedentary life), perineal trauma (pelvic floor muscle tenderness, sitting position, traumatic sports for perineum, constrictive clothing; Gallo, 2014) (Figure 2). These risk factors should be avoided and lifestyle adjustment advised, such as changes in diet and sexual habits. Some of these changes cannot be easily achieved for individuals without communication and support from sexual partners and family. Community and social support are sometimes required, for instance, in jobs involving long spells of sitting to allow a period of active walking. Nowadays, the digital era provides more channels than ever before for communication. New apps for mobile devices are anticipated in the near future for monitoring selected medical treatment of CP/CPPS and following up. This will extend an effective treatment schedule of CP/CPPS and easily accessible assessment beyond the doctor’s office.

In conclusion, the perspectives on the diagnosis, treatment, and prognosis of CP/CPPS are illustrated and updated comprehensively. These insights from literature and the authors’ experience will hopefully reduce some levels of frustration for urology care providers who are dealing with CP/CPPS in their daily work and help move toward a better management of patients with CP/CPPS. Ongoing research and accumulated clinical evidence will further the knowledge of CP/CPPS and reveal its etiology and pathophysiological mechanism in the future.
